# The Use of the Humanized Mouse Model in Gene Therapy and Immunotherapy for HIV and Cancer

**DOI:** 10.3389/fimmu.2018.00746

**Published:** 2018-04-20

**Authors:** Mayra A. Carrillo, Anjie Zhen, Scott G. Kitchen

**Affiliations:** Department of Medicine, Division of Hematology and Oncology, University of California Los Angeles, Los Angeles, CA, United States

**Keywords:** HIV, cancer, humanized mice, gene therapy, immunotherapy, T cell receptor, chimeric antigen receptor, hematopoietic stem cells

## Abstract

HIV and cancer remain prevailing sources of morbidity and mortality worldwide. There are current efforts to discover novel therapeutic strategies for the treatment or cure of these diseases. Humanized mouse models provide the investigative tool to study the interaction between HIV or cancer and the human immune system *in vivo*. These humanized models consist of immunodeficient mice transplanted with human cells, tissues, or hematopoietic stem cells that result in reconstitution with a nearly full human immune system. In this review, we discuss preclinical studies evaluating therapeutic approaches in stem cell-based gene therapy and T cell-based immunotherapies for HIV and cancer using a humanized mouse model and some recent advances in using checkpoint inhibitors to improve antiviral or antitumor responses.

## Introduction

Humanized mice have emerged as an invaluable tool in providing a model system that enables researchers to study the human immune system and its development and function/dysfunction *in vivo* (1). The identification of the severe combined immunodeficiency (*Prkcd^sci^*^d^ or SCID) mouse provided the cornerstone of the development of the humanized mouse model by allowing the xenoengraftment of human (hu) cells [specifically, human peripheral blood lymphocytes (PBLs)] without mouse immune system-mediated rejection (known as the hu-PBL SCID model) (2). This allowed limited examination of components of the human immune system in a manipulatable model system. Further development occurred with the engraftment of SCID mice with human fetal thymus and liver tissue, which is implanted under the kidney capsule of the animals (termed the SCID-hu mouse) (3, 4). The fetal liver tissue provided the hematopoietic cells and the thymus tissue provided the stromal elements to facilitate the engraftment and development of a functional human thymus in these animals. This allowed the closed examination and long-term engraftment of human hematopoietic tissue *in vivo*. Humanized mouse model development rapidly expanded with the identification and breeding of immunodeficient strains of mice that facilitated a greater engraftment of human cells. SCID mice have been crossed with other mouse strains, such as the nonobese diabetic (NOD) mouse to generate NOD/SCID mice that have defects in innate and adaptive immunity (5). Other mice that have been crossed to SCID mouse strains include those that have genetic mutations in the Rag1, Rag2, or the IL-2 receptor common gamma chain (IL2rγ) genes to generate new strains of immuno-incompetent mice which allow greater human cell and tissue engraftment, particularly the tissues and cells that have a high hematopoietic potential (6). The NOD/SCID and NOD/SCID/IL2rγ-knockout (NSG) strains have been used to generate one of the more recent humanized mouse models that has shown to have the most robust human immune system engraftment, providing long-term human hematopoietic stem/progenitor cell (HSPC) engraftment and functional multilineage hematopoietic differentiation. This model facilitated the engraftment of human CD34^+^ HSPCs in the bone marrow of the animals and subsequent multilineage hematopoiesis, including B cell production and limited T cell development [termed the CD34-humanized mouse (7)]. More robust T cell reconstitution, which provides a more relevant model for HIV infection and the study of T cell immunity (8), was subsequently developed and involved the intravenous injection of autologous CD34^+^ human hematopoietic cells from fetal liver tissues, which engraft in the bone marrow (B), along with the transplantation of human fetal liver (L) and thymus (T) tissue under the kidney capsule of the mice, which forms a recapitulated human thymus [known as the bone marrow–liver–thymus (BLT) mouse] (9, 10). New mice strains, such as NOD-SCID IL2Rγnull/IL-3/GM-CSF(NSG-SGM3), are also being adopted for constructing BLT mice for better differentiation of myeloid cells or cancer engraftment (11, 12). Overall, immune-incompetent mouse strains can be humanized by either the transplantation of human peripheral blood mononuclear cells (PBMCs), the transplantation of human HSPCs, or the engraftment of human fetal tissue and HSPCs (Table 1). Among them, the humanized BLT mice are the most robust model in supporting multilineage human immune system development (13). The development of humanized mouse models has been extensively reviewed in Ref. (6, 14, 15) and been utilized in preclinical studies that revealed important discoveries in several fields of research (1).

**Table 1:** Engraftment of human immune system in the most commonly used immunodeficient mouse models.

Common engraftment method of human cells	Common immunodeficient strains used	Characteristics of the reconstituted human immune system	Source and references
Injection of peripheral blood mononuclear cells	Nonobese diabetic (NOD).CB17-*Prkdc^scid^* (NOD-SCID)	Engraftment of T cells, rapid GVHD development	Jackson Laboratory (2)
Injection of HSCs	NOD.Cg-*Prkdc^scid^IL2rg^tm1WjN^/Sz* (NSG)	Multiple hematopoietic lineages including T and B cells, APCs, and NK cells	Jackson Laboratory (7)
Implantation of fetal liver and thymus tissue	NOD-SCID NSG	Robust thymocyte development, thymocytes educated on autologous thymic epithelium, minimal development of peripheral immune system	Jackson Laboratory (3, 4)
Implantation of fetal liver and thymus tissue and injection of autologous HSCs	NSG NOD-SCID NOD.129S7 (B6)-*Rag1^tm1Mom^IL2rg^tm1WjN^/Sz* (NRG) B6.129 (Cg)-*Rag2^tm1Fwa^Cd47^tm1FpI//2rgtm1WjI/J^* (TKO-C57BL6)	Complete human immune system, human leukocyte antigen-restricted T cells, mucosal immune system, delayed GVHD	Jackson Laboratory (14, 16–18)

In particular, HIV researchers have taken advantage of the humanized mouse model to better understand the pathogenesis of the infection and to examine novel therapeutic strategies to treat and possibly eradicate infection (19). Relatively early in the use of these types of humanized mice, researchers used the SCID-hu mouse as a platform to design and test a gene therapy approach for the treatment of HIV infection. Human HSPCs were transduced with a retroviral vector expressing a reporter gene and were then injected into the human thymus organoid to evaluate the differentiation and development of mature cells carrying the transgene reporter *in vivo* (20, 21). These studies formed the basis of the development of this approach to protect cells from HIV infection in what was the largest phase II gene therapy trial to that date (22). This sets the stage for the forward progression of other types of HSPC-based gene therapy research involving the development of lentiviral vectors expressing anti-HIV components that result in HIV-resistant immune cells *in vivo* in humanized mice (23–27). Results for some of these studies enabled stem cell-based gene therapy clinical trials that are currently ongoing (ClinicalTrials.gov Identifier: NCT01734850). Thus, studies such as these performed in humanized mice illustrate the utility of testing new stem cell-based gene therapy approached in humanized mice and highlight the potential therapeutic efficacy and safety of engineering such aspects as HIV resistance through the genetic modification of HSCs with anti-HIV genes (28).

Currently, humanized mouse models are being highly utilized to study human diseases and develop novel therapeutic approaches that can potentially be translated into clinical trials as described above. HIV and cancer are two research fields that have been taking advantage of the humanized mouse model to study stem cell- and T cell-based immunotherapy approaches to treat these chronic diseases. In this review, we highlight important studies using the humanized mouse model in stem cell- and T cell-based immunotherapy using highly potent transgenic T cell receptors (TCRs) and chimeric antigen receptors (CARs). We also discuss utilizing checkpoint inhibitors to overcome common immunosuppression mechanisms used by both diseases that promote disease progression and persistence.

## Peripheral Cell-Based Immunotherapy Modeling in Humanized Mice

### Transgenic TCRs in Humanized Mice

One of the earliest attempts for treating HIV through an immunotherapy-based approach using peripheral T cells was to isolate HIV-specific CTLs from HIV patients, expand *ex vivo*, and infuse them back into the patients (29–32). However, these studies demonstrated that this approach had very little impact on antiviral efficacy in treated individuals. There are current attempts to improve the efficiency of this approach through the “redirection” of peripheral T cells to target HIV infection through the genetic modification of cells with HIV-specific, molecularly cloned TCRs [for review on transgenic TCRs, see Ref. (33, 34)]. Proof of principle studies were conducted in the humanized mouse model wherein Joseph et al. produced a lentiviral vector encoding the TCR that recognizes the HIV-1 gag epitope SL9, which elicits a potent antiviral response by CTLs carrying the SL9-specific TCR (35). Using the SCID-hu mouse model, transduced CD8^+^ T cells carrying the SL9-specific TCR were co-injected with human leukocyte antigen (HLA)-matched HIV-1-infected PBMCs and tested for *in vivo* suppression of HIV-1. Isolated spleens of the mice treated with transduced HIV TCR CD8 T cells showed no signs of HIV-1-infected PBMCs; thus, peripheral CD8^+^ T cells modified with this potent anti-HIV TCR were capable of controlling and clearing HIV-1 infection *in vivo*. Although TCR-based immunotherapy has been shown to be effective in nonhumanized mouse models (36–38), there are rising safety concerns with using cloned TCRs in adoptive immunotherapy because of the possibility of exogenous TCR mispairing with an endogenous TCR chain, generating a new TCR that can have lethal off-target toxicity (39, 40). However, other studies conducted in humanized mice suggest that this may not be a significant issue (see below).

### CAR-Based Immunotherapy in Humanized Mice

An ever-present issue with the use of molecularly cloned TCRs in therapy is that they have to be used in HLA-matched individuals, lessening their potential use to a limited number of people. CARs, which combine antigen-recognizing, HLA-independent extracellular domains with the TCR-zeta chain intracellular signaling domain, broaden these molecules’ potential use as a T cell redirection/engineering therapeutic approach [for a review on CAR T cell design, see Ref. (41)]. There have been numerous preclinical studies and clinical trials that have tested or are currently testing the effectiveness of CAR T cell therapy against certain cancers, reviewed in Ref. (42). In many preclinical studies, humanized mice were used to test the antitumor efficacy of various CAR designs: for example, second- or third-generation CARs which contain immune-enhancing costimulatory domains (43–45). Humanized mice can also be used to study the effect of combination therapy with CAR T cells and antibody-targeting immune checkpoint inhibitors such as PD-1 and CTLA-4 (46). A combinatorial therapeutic approach using CAR T cells and an immune checkpoint inhibitor has recently been studied in a humanized mouse model of metastatic clear-cell renal cell carcinoma (47). These CAR T cells targeting human anti-carbonic anhydrase are also equipped to secrete human anti-programmed death ligand 1 (PD-L1) antibodies to overcome checkpoint inhibition mediated by PD-1 and PD-L1 interactions. This approach to immune-checkpoint blockade resulted in an enhanced antitumor efficacy compared to mice treated with CAR T cells alone. Continuous efforts to study the behavior of CAR T cells *in vivo* using humanized mice can provide important understandings into overcoming the immunosuppressive properties of the tumor microenvironment.

With the success of CAR T cell therapy against B cell malignancies, HIV researchers are revisiting the CAR T cell approach for the treatment of HIV infection (48–50). Very recently, peripheral anti-HIV CAR T cells have been tested for antiviral efficacy using a humanized mouse model of HIV infection (51). The study’s approach was to redesign a CD4-based CAR vector used previously in clinical trials to augment expression and CAR T cell performance. Anti-HIV CAR T cells that contained the costimulatory 4-1BB domain outperformed those that contained the CD28 costimulatory domain in reducing viral rebound after ART treatment and prolonged persistence *in vivo* in the absence of antigen. Thus, opposed to the minimal clinical efficacy seen with the first-generation CD4-based CAR, newer generation of anti-HIV CARs can potentially have a more promising outcome in clinical trials. Future studies using humanized mouse models of HIV infection can provide more information on differences in anti-HIV responses and the clearance of HIV infection *in vivo* using anti-HIV CAR T cells containing different combinations of costimulatory domains.

## Stem Cell-Based Gene Therapy in Humanized Mice

Recent developments of new humanized mouse models have opened opportunities in efforts to modify human stem cells to generate an immune system designed to mount a more efficient, targeted immune response against a specific pathogen or a disease. Humanized mice are being employed to test the therapeutic efficacies of stem cell-based gene therapies involving the modification of HSPCs with potent antigen-specific TCRs and CARs, and engineering a human immune system equipped to specifically target HIV or cancer antigens *in vivo*. Below, we discuss key studies that have utilized the humanized mouse model system for stem cell-based therapy for HIV and cancer.

### Stem Cell-Based Gene Therapy Using TCRs Against HIV and Cancer

To enhance the immune response to HIV infection, studies have used HSPCs to introduce HIV-specific TCRs into immunodeficient mice to reconstitute a human immune system that contains a population of T cells carrying an HIV-specific TCR. The testing of this concept initially utilized the SCID-hu mouse model (52). CD34^+^ HSPCs were isolated from a human fetal liver, transduced with a molecularly cloned anti-HIV TCR, and transplanted into irradiated HLA-matched SCID-hu mice. This resulted in the generation of mature CD8^+^ T cells carrying the transgenic anti-HIV TCR. These anti-HIV TCR^+^ T cells were functional in response to peptide stimulation *ex vivo*, differentiating into effector cells, producing interferon (IFN)-gamma, and lysing targeted cells. To test the functionality of anti-HIV TCR^+^ T cells generated from transduced HSCs *in vivo*, a follow up study used the NSG strain mouse that is engrafted with human liver/thymus and injected with transduced fetal liver CD34^+^ cells. Using this NSG-CTL mouse model, the injected transduced HSCs were able to differentiate into mature human CD8^+^ T cells carrying the transgenic anti-HIV TCR (16). More importantly, anti-HIV TCR^+^ CD8 T cells were found to migrate into multiple tissues including the spleen, bone marrow, and the implanted human thymus. Following an HIV-1 challenge into these mice, these anti-HIV TCR^+^ CD8 T cells were able to suppress viral load at 2 weeks and 6 weeks post infection in the peripheral blood. In addition, mice carrying the anti-HIV TCR T cells were protected against CD4 T cell depletion and had lower levels of infected cells by 6 weeks post infection. Other key outcomes observed in this study were the reduced viral burden in anti-HIV TCR mice in lymphoid tissues and the expansion and differentiation of anti-HIV TCR^+^ T cells in response to an active HIV infection. These studies using two different humanized mouse models showed the feasibility and therapeutic potential of modifying HSCs with a potent anti-HIV TCR to produce a functional antiviral immune response to HIV.

Investigators have turned to the humanized mouse model to test the proof of principle of this type of stem cell-based gene therapy against cancer. Similar to the HIV-based studies, stem cell-based gene therapy for cancer is also being examined as a potential therapeutic strategy to provide a long-lasting immune surveillance against tumor cells using human HSPCs modified with an antitumor TCR (53). Using the BLT-humanized mouse model, Vatakis et al. transplanted HSPCs modified with a HLA-A*0201-restricted anti-melanoma TCR (54, 55). The transduced HSPCs were able to differentiate and produce high levels of naïve CD8^+^ T cells carrying the anti-melanoma TCR. Upon challenging these mice with HLA-matched tumors, mice treated with anti-melanoma TCRs were able to control tumor growth, and in some mice, clear the tumor compared to control mice carrying nonmodified T cells. Further analysis on the functionality of these anti-melanoma-specific T cells showed that they can differentiate into different subsets of effector and memory phenotype and infiltrate into tumors. Moreover, analysis of the bone marrow of these mice carrying transgenic HSCs showed continued expression of the integrated vector in isolated bone marrow samples. Thus, transgenic HSPCs can repopulate the bone marrow and provide a long-lasting supply of modified mature immune cells, including T and natural killer (NK) cells, directed against a specific pathogen. Other studies have also utilized the CD34-humanized mouse model in examining stem cell gene therapy using candidate antitumor specific TCRs which exhibited similar and new informative outcomes (56–58). In particular, these studies found that the introduction of the TCR transgene in HSPCs could inhibit endogenous TCR rearrangement in T cells (56, 57, 59). This is an important discovery as it can overcome the potential of off-target toxicities from transgene expression and endogenous TCR chains rearrangement and alpha and beta chain receptor mixing. Hence, humanized mouse models enabled investigators to study the development and dynamics of an immune system with unlimited replenishment of immune cells carrying a disease-specific receptor which can provide key aspects of its therapeutic potential in clearing a persistent infection or a disease.

### Stem Cell-Based CAR T Cell Studies in HIV and Cancer

To test the safety and efficacy of a stem cell-based CAR approach in HIV infection, Zhen et al. used the BLT-humanized mouse model and modified HSPCs with a lentiviral vector expressing an anti-HIV CD4-based CAR to determine whether this can result in the generation of mature anti-HIV CAR^+^ CTLs (17). This anti-HIV CAR is based on utilizing the HIV receptor CD4 molecule that is fused to an internal TCR-signaling domain (60). Stem cells from fetal liver were modified with anti-HIV CAR-expressing lentiviral vector and infused into NSG mice transplanted with fetal liver and thymus. Investigators observed subsequent maturation of CAR^+^ T cells, NK cells, B cells, and myeloid cells *in vivo*. In addition, cells carrying the CAR-expressing lentiviral vectors were protected from HIV infection by coexpressing protective anti-HIV shRNAs and were able to functionally suppress HIV replication *in vivo* through CTL activity. Also, similar to the TCR-modified HSPC-based studies, developing T cells carrying the anti-HIV CAR receptor can successfully go through positive selection in a human thymus, and the expression of the anti-HIV CAR resulted in the suppression of endogenous TCR rearrangement. This observation that developing T cells expressing an anti-HIV CD4-based CAR suppressed endogenous TCR rearrangement suggests that the CD4-based CAR can act as the sole natural TCR during development. This could be a beneficial trait in the long term, as emerging T cells expressing CD4-based CARs will be specific to HIV antigen and chances of off-target activation will be minimal. A similar approach was also done examining the development of CD19CAR-expressing cells in the CD34-humanized mouse model (61, 62). They found that the introduction of a lentiviral vector expressing either a CD19CAR or a second-generation CD19CD28CAR into HSPCs and engrafting into NSG mice led to the differentiation of different hematopoietic lineages expressing CAR including T cells, B cells, and myeloid cells and produced potent antitumor responses in the CD19CD28CAR-treated mice (61, 62). It remains to be seen if the therapeutic effects of stem cell-based CAR T cell therapy performed on humanized mice will be translated into human clinical trials.

## PD-1 and IFN-I Blockade Therapy for HIV and Cancer

While humanized mice have been useful in the examination of human immunotherapeutic approaches involving gene therapies, their use in examining antiviral or antimalignancy responses and immunotherapies is at a relatively nascent stage. More sensitive immune-based assays and improvements in humanized mice now allow the examination of antitumor and antiviral immune responses and show great promise in the development of novel immunotherapies to treat these conditions. In recent studies, humanized mouse models were used to examine the effects of blocking key immune and antiviral factors in chronic HIV infection. Chronic viral infections can persist by upregulating immune checkpoint receptors that can functionally compromise virus-specific T cells and prevent them from clearing the infection (63). HIV infection has been shown to upregulate T cell exhaustion markers that enable the virus to chronically persist, which includes PD-1, Tim-3, LAG-3 among others (64–69). To investigate whether T cell exhaustion can be reversed and rescue function in exhausted T cells, these recent studies closely examined immune factors in chronically HIV-infected mice and found elevated PD-1 levels on T cells, similar to that seen in infected individuals. These chronically infected mice were treated with an antibody that blocks the PD-1/PD-L1 pathway and found reduced viral loads and increased CD4^+^ and CD8^+^ T cell levels (70, 71). In addition, PD-L1 blockade increased the percentages of naïve and central memory T cells and increased Th1 cytokines IFN-gamma and IL-12 during treatment (70). Thus, blocking the PD-1/PL-1 pathway during chronic HIV leading to reduced viral loads has now been shown in two different humanized mouse models and supports results seen in a study applying PD-1 blockade during chronic SIV infection in a macaque model, which reduced SIV levels (72). It remains to be seen whether PD-1/PD-L1 blockade can have clinical success in antiviral therapy in chronically HIV-infected individuals as it has already been observed in individuals treated for human cancer (73–75). PD-1 blockade treatment for cancer therapy has been shown to have therapeutic benefits in patients with certain types of malignancies (76). Recently, preclinical studies have utilized humanized mice either transplanted with human CD34^+^ HSPCs (HuNSG) or mice containing a double knockout of MHC class I or class II (NOG-dKO) to show the therapeutic potential of utilizing PD-1 blockade for cancer therapy (77, 78). These studies highlight the usefulness of humanized mice to study not only the antitumor effects of anti-PD-1 blockade but also the human immune responses to human tumors, as these studies revealed significant tumor growth suppression and antitumor CD8^+^ T cell responses following PD-1 blockade treatment.

Hyper-immune activation is a hallmark of chronic HIV infection, and arising evidence is suggesting that chronic type I (IFN-I) is driving this continuous immune activation that may be leading to disease progression (79). To investigate the role IFN-I plays in driving chronic HIV infection, investigators have turned to BLT-humanized mouse models of HIV infection to study this (80, 81). In the study by Zhen et al., after establishing a chronic HIV infection, blocking IFN-1 signaling using an anti-interferon alpha receptor 2(IFNR2)-blocking antibody resulted in a decreased immune activation, a decreased expression of T exhaustion markers and reversal of T cell exhaustion, and reduced plasma viral loads. In addition, treatment with the anti-IFNR2-blocking antibody in combination with ART resulted in a rapid viral suppression and reduced viral reservoirs. Cheng et al. found similar results using IFNR1-blocking antibody in combination with ART treatment throughout their study (80). These results shed light on the role IFN-I signaling plays during chronic HIV infection in maintaining chronic immune activation and T cell exhaustion that leads to uncontrolled HIV infection *in vivo*. Findings from these and future studies may lead to the application of IFN-I blockade treatment in combination with ART during chronic HIV infection that could alleviate residual immune activation and reduce viral reservoirs in HIV-positive individuals. It remains to be seen whether IFN-I blockade will have a beneficial antitumor efficacy during tumor progression since IFN-I is important in inducing antitumor responses such as promoting CD8 T cell priming. However, continuous IFN-I signaling can also have immunosuppressive properties that may play a role in promoting tumor growth (82). It has been recently shown that continuous IFN signaling drives PD-L1-dependent and -independent resistance to radiation therapy and checkpoint blockade, and blocking IFN-I signaling restores tumor cell response to checkpoint blockade treatment (83). Whether IFN-I blockade treatment can restore response to treatment in tumors that are resistant to PD-1 blockade or other immune checkpoint blockade in a humanized mouse model of cancer remains to be determined.

## Future Directions

Although humanized mice have been an essential tool in several fields of research to better understand the mechanisms of disease progression and develop therapeutic strategies, these mouse models do come with their own limitations that need to be addressed to create more optimized models that will fit the needs of each research field (84). Currently, SCID mice engrafted with human PBMCs develop graft-versus-host disease (GVHD) within 4 weeks of engraftment, limiting the time of experimentation to just a few short weeks. The humanized BLT mouse model also has its own limitations for use. BLT mice can have poor B cell development, limited antibody class switching following activation, and lymphocyte homing in lymph nodes and germinal centers, limiting their antibody responses. In addition, these mice also typically develop a GVHD-like condition after around 20 weeks post engraftment of fetal tissue and HSCs, putting a limitation on the duration of a given study (84, 85). Therefore, there is a pressing need to develop new mouse strains with genetic properties that will eliminate the generation of this GVHD-like condition. Recently, a new modification of the BLT mouse model was made by transplanting fetal thymus, liver, and autologous CD34^+^ HSCs into a C57BL/6 mouse strain that contain a triple knockout of Rag2, IL-2Υc, and CD47 genes (TKO-BLT) (18). These mice were observed to be healthy with no signs of GVHD for 45 weeks post transplantation, which is months longer than that of the current BLT models. In addition, they retained high reconstitution of human cells throughout the 45 weeks. They also found this model to establish HIV latency, respond well to orally fed and subcutaneously injected ART treatment, and upon ART interruption, can generate rapid viral rebound. Thus, this new humanized TKO-BLT mouse model can provide an extended duration of a variety of studies that will be useful for addressing issues requiring longer periods of infection or disease progression.

Because of the variety of humanized mouse models currently available, it is important for investigators to be knowledgeable on the different mouse models and which one will be the more appropriate model to answer the questions they are investigating. Differences in the background mutations of the immunocompromised strains can have an impact on the engraftment of human cells and the development of peripheral lymph nodes and germinal centers (14). Therefore, results using humanized mice must be carefully interpreted. It is also important to include proper controls, particularly for immune-based studies, such as uninfected and unmanipulated animals, to control for any potential changes/interference by GVHD or specific effects pertaining to the individual tissues.

Humanized mouse models are also currently being improved upon for cancer research (86). Cancer therapy studies evaluating the immune response to tumors would benefit from a humanized BLT model where the human reconstituted immune system is compatible with the transplanted tumor tissue. One possibility will be to acquire HSPCs from a patient and transplant autologous tumor cells or HLA-matched tumor cells into the mice. This will generate a closer representative of the patient’s antitumor response without the interference of alloreactive T cells resulting from the mismatch of the reconstituted immune system and engrafted tumor cells. Further advances in generating humanized mouse models that overcome current limitations will be highly beneficial for HIV and cancer researchers to advance stem cell-based gene therapy, T cell immunotherapy, and other immunological studies such as T cell exhaustion and tumor immunosuppressive microenvironment for eradicating HIV and cancer.

## Author Contributions

MC, AZ, and SK contributed equally to the preparation of this manuscript.

## Conflict of Interest Statement

The authors declare that the research was conducted in the absence of any commercial or financial relationships that could be construed as a potential conflict of interest.
